# The Gracilis Muscle Reappraised: An Integrative Synthesis of Anatomy, Embryology, Imaging, and Surgical Applications

**DOI:** 10.3390/jcm15082988

**Published:** 2026-04-15

**Authors:** Ingrid C. Landfald, Paloma Aragonés, Dawid Pilewski, Łukasz Olewnik

**Affiliations:** 1 Department of Clinical Anatomy, Mazovian Academy, 09-402 Płock, Poland; I.landfald@mazowiecka.edu.pl (I.C.L.); d.pilewski@mazowiecka.edu.pl (D.P.); 2 Department of Orthopedic Surgery, Faculty of Medicine, Universidad Complutense de Madrid, 28040 Madrid, Spain; paloarag@ucm.es

**Keywords:** obturator nerve, medial circumflex femoral artery, motor points, perforators, MR neurography, computed tomographic angiography, ultrasound, free functional muscle transfer

## Abstract

**Background and Objectives:** Fragmented anatomical, imaging, and surgical accounts of the gracilis muscle hinder reproducible reporting and operative planning. We aimed to integrate prior systems into an Integrated Gracilis Framework (IGF)—an integrative synthesis, not a new classification—that harmonizes terminology, defines imaging correlates/pitfalls, and links morphology to surgical decisions. **Methods:** Integrative narrative review (January 1900–October 2025) of PubMed/MEDLINE, Scopus, and Web of Science covering vascularization (pedicles, perforators), innervation (motor points/segments), imaging (ultrasound, MRI, MR neurography, CTA/MRA), and clinical applications (facial reanimation, elbow flexion, perineal and breast reconstruction). Two reviewers screened/extracted with consensus adjudication. Searches were restricted to English or records with reliable English-language summaries. **Results:** IGF consolidates morphological variants, motor-point/segmental innervation, and pedicle/perforator patterns with imaging correlates and common pitfalls. It provides a crosswalk mapping historical systems to IGF and a proposed preoperative workflow (anatomy → imaging → harvest → neurotization) for structured planning and reporting (proposed framework; not prospectively validated). We summarize considerations for free/functional gracilis in facial reanimation and elbow-flexion, and for pedicled/free myocutaneous or perforator flaps in perineal and breast reconstruction. **Conclusions:** IGF offers a standardized language and decision scaffold to improve study comparability and transparency in operative reporting; as a nonvalidated synthesis, it requires systematic validation through cadaver–imaging correlation and prospective surgical cohorts.

## 1. Introduction

The gracilis muscle (GM) is a long, superficial, strap-like muscle situated in the medial compartment of the thigh. It originates from the inferior pubic ramus and inserts onto the medial surface of the tibia, forming part of the pes anserinus together with the sartorius and semitendinosus tendons [1,2]. Functionally, it contributes to hip adduction, knee flexion, and internal rotation of the leg [1,3]. The GM is typically supplied by branches of the medial circumflex femoral artery (MCFA) and/or the deep femoral artery (DFA) [1,3], and is innervated by the anterior branch of the obturator nerve arising from the ventral rami of L2–L4 [4].

The GM has considerable value in reconstructive surgery due to its functional redundancy, reliable vascularisation, and favourable donor-site profile. It is classified as a Type II muscle flap according to the Mathes and Nahai system, with one dominant vascular pedicle and several minor pedicles [3,5]. These properties have led to its widespread use in dynamic facial reanimation, upper limb reinnervation, lower limb defect coverage, and breast reconstruction procedures, including the transverse upper gracilis (TUG) and diagonal upper gracilis (DUG) flaps [6,7].

Despite its generally predictable gross anatomy, the GM displays notable variation in muscle belly morphology, tendon configuration, vascular supply, and neural architecture [4,8,9,10]. Such variability can significantly influence flap viability, pedicle dissection complexity, and functional outcomes. For example, multiple vascular pedicles may necessitate modification of the dissection approach, short tendon length may limit reach in free transfers, and dual innervation can enable functional muscle splitting in specialised reconstructive procedures [3,4,9].

Several classification systems addressing GM anatomy have been proposed; however, most focus on isolated features such as tendon insertion patterns [11], vascular supply [3], or neural branching [4,9]. This fragmentation limits clinical applicability, as surgeons require an integrated framework to support preoperative imaging interpretation and structured operative planning.

Rather than proposing a novel classification, we present an Integrated Gracilis Framework (IGF) that harmonizes prior anatomical, imaging, and surgical systems into a single, practical crosswalk. IGF is explicitly an integrative synthesis derived from published work, offered to standardize terminology and decision points across domains; it is a proposal requiring external validation [12]. The IGF is a proposed integrative framework (crosswalk) intended to structure reporting and support preoperative planning; it has not yet been prospectively validated as a clinical decision rule.

The aim of this review is to consolidate anatomical, radiological, and clinical knowledge of the GM; present the IGF in comparison with existing systems; and demonstrate its utility in preoperative mapping and surgical reconstruction through integrated anatomical–radiological correlations.

**State of the art & working hypothesis.** Existing frameworks typically treat tendon/morphology, vascular supply, and innervation in isolation, which limits clinical translatability and concordance with imaging. We hypothesize that an imaging-traceable, domain-integrated scaffold—the IGF—will improve reporting consistency and preoperative planning relative to domain-specific descriptions. **Objectives.** Our objectives were to: (1) consolidate anatomical, imaging, and surgical evidence on the GM; (2)present IGF explicitly as an integrative synthesis/crosswalk of prior systems; and (3) demonstrate how IGF supports preoperative mapping, flap harvest, and neurotization planning steps (proposed framework; not prospectively validated).

## 2. Methods

**Design.** Systematized integrative review with narrative synthesis. We used a PRISMA-style flow diagram to transparently document record identification, screening, and inclusion, without claiming full PRISMA compliance.

**Protocol and registration.** No preregistered protocol; the review questions, eligibility criteria, and extraction fields were defined a priori.

**Information sources.** PubMed/MEDLINE, Scopus, and Web of Science were searched from January 1900 to October 2025. Reference lists of key articles were hand-searched; forward citation tracking was performed.

**Search strategy.** Database-specific Boolean strings were used and refined iteratively.

- **PubMed/MEDLINE:** ((“gracilis muscle”[MeSH] OR gracilis[All Fields] OR “gracilis flap”[All Fields]) AND (anatom*[All Fields] OR vascular OR perforator* OR “motor point*” OR innervation OR “magnetic resonance neurography” OR MRI OR ultrasound OR CTA OR MRA OR reconstruction OR “free functional muscle transfer” OR “facial reanimation” OR “elbow flexion”))
- **Scopus (TITLE-ABS-KEY):** gracilis AND (anatom* OR vascular OR perforator* OR “motor point*” OR innervation OR “magnetic resonance neurography” OR MRI OR ultrasound OR CTA OR MRA OR reconstruction OR “free functional muscle transfer” OR “facial reanimation” OR “elbow flexion”)
- **Web of Science (TS):** gracilis AND (anatom* OR vascular OR perforator* OR “motor point*” OR innervation OR “magnetic resonance neurography” OR MRI OR ultrasound OR CTA OR MRA OR reconstruction)

**Eligibility criteria:**

**Inclusion:** Human studies reporting **primary** anatomical, imaging, or surgical data on the gracilis muscle or its vascular/neuronal supply; clinical uses including facial reanimation, elbow-flexion reconstruction, and perineal/breast reconstruction.

**Exclusion:** Editorials/opinions and narrative reviews without new data; animal/in vitro models without human translation; duplicate cohorts (most complete retained); case series < 3 without extractable anatomical/imaging parameters; records without full text or a reliable English-language summary; studies outside anatomy/imaging/surgical use of gracilis.

**Language restrictions (justification).** Searches were restricted to English-language articles or records with **reliable English-language summaries** to minimize extraction errors and reflect the language used in clinical settings that apply IGF. Potential omission of historical non-English reports is acknowledged under **Limitations**.

**Study selection and data extraction.** Two reviewers independently screened titles/abstracts and full texts; disagreements were resolved by consensus. Predefined forms captured morphology (belly/tendon, pes anserinus), vascular pedicles/perforators (origin, caliber, length), innervation (motor points/segments), imaging protocols/findings (ultrasound, MRI/MR neurography, CTA/MRA), and surgical variables (indication, harvest, inset orientation, neurotization, outcomes/complications).

**Quality appraisal (methodological variability).** Given the heterogeneity of anatomical, imaging, and clinical study designs, we performed a qualitative appraisal rather than a formal risk-of-bias scoring. Two reviewers assessed key sources of methodological variability (study design and sample size; anatomical technique and definitions; imaging modality/protocol and interpretation; outcome ascertainment and reporting completeness) and used this appraisal to contextualise the strength and generalisability of the narrative synthesis.

**Synthesis approach.** Findings were narratively integrated into the **Integrated Gracilis Framework (IGF)** domains (morphology, vascularization, innervation), linked to imaging correlates and pitfalls, and organized into a proposed preoperative workflow (anatomy → imaging → harvest → neurotization) to structure synthesis across domains (proposed framework; not prospectively validated). Historical nomenclatures were mapped to a single **crosswalk**.

**Transparency.** No generative AI tools were used for content drafting, data generation, analysis, or scientific interpretation.

**Figure 1:**
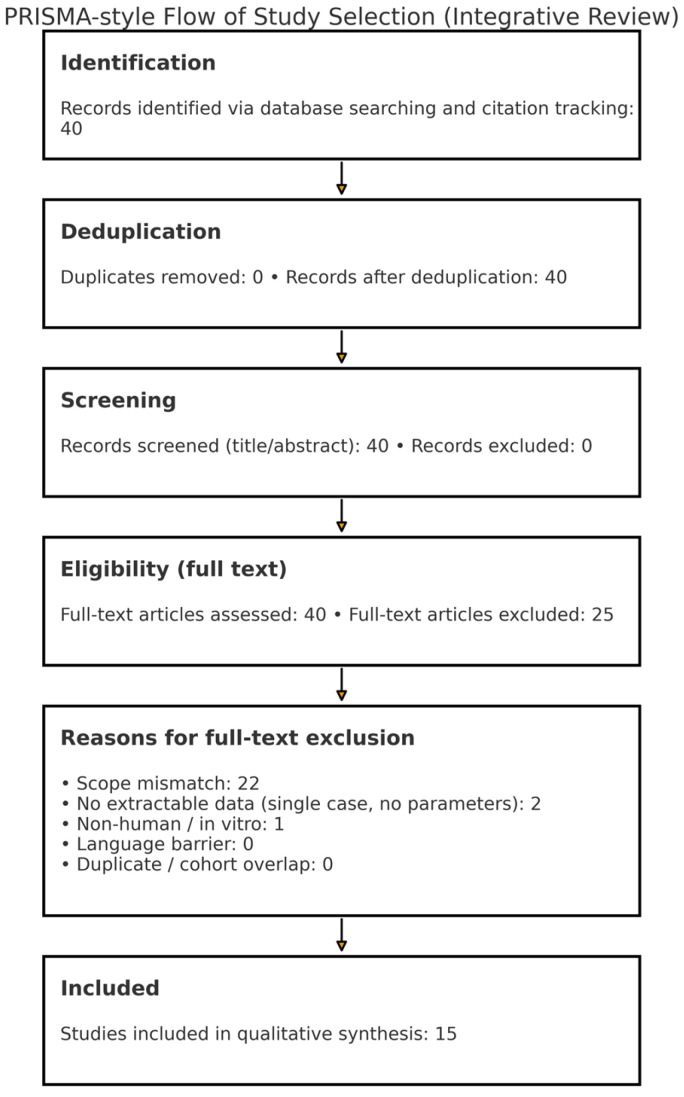
PRISMA-style flow of study selection. Counts reflect the corpus as of **October 2025**.

**Reporting aid.** Study selection is summarized in Figure 1 **(PRISMA-style flow)**.

## 3. Embryology of the Gracilis Muscle

### 3.1. General Embryological Development

The GM develops from the ventral (adductor) muscle mass derived from hypaxial myotomes that migrate into the lower-limb bud during weeks 5–8 of gestation. Subsequent compartmentalisation and splitting of the ventral mass establish the medial thigh musculature, including the GM [13,14,15,16].

### 3.2. Myogenic Differentiation and Patterning

Myogenic differentiation and spatial patterning in the limb are regulated by conserved signalling pathways, including Shh, Wnt and FGF cascades, which coordinate myoblast proliferation, migration and muscle splitting [15,16,17]. Minor deviations in separation of the ventral muscle mass or tendon remodelling may plausibly result in accessory slips, atypical distal morphology or partial bifurcation, providing a developmental basis for recognised adult variants [13,15,16].

### 3.3. Development of Innervation

Innervation is established as motor axons from the lumbosacral plexus extend into the limb bud, with the GM typically supplied by the anterior division of the obturator nerve (L2–L4) [1,16]. Subtle alterations in axonal guidance and peripheral nerve patterning may underlie reported variations, including dual innervation or anomalous neural communications [12,18,19].

### 3.4. Vascular Development and Surgical Relevance

Vascularisation follows ingrowth of primitive axial arterial patterns into the limb bud and subsequent selective remodelling, producing the mature arterial arrangement supplying the GM, most commonly via branches associated with the deep femoral system [15,16]. Developmental variability and later remodelling may explain multiple pedicles or atypical arterial origins, which are directly relevant for flap viability and pedicle dissection strategy [4,8,9,12].

## 4. Morphological Variations of the Gracilis Muscle

### 4.1. Muscle Belly Variants

The GM typically presents as a slender, superficial muscle belly along the medial thigh, with a single strap-like morphology in ~98% of cases [1,2], enabling straightforward dissection.

Double-headed gracilis (“gracilis biceps”) is a rare (~2%) but clinically significant variant, comprising two distinct heads: one from the pubis and one from the ischial ramus. If unrecognized, it may be mistaken for another structure; conversely, it can provide additional tissue for reconstruction [3].

Accessory muscle slips, though rare, may extend toward the fascia lata or gastrocnemius, increasing dissection complexity or offering extra graft material [2].

Radiological detection relies on axial MRI (T1-weighted or PD) and high-frequency ultrasound, which visualize separate bellies or diverging slips from the main GM body [1,2,11,20].

**Figure 2:**
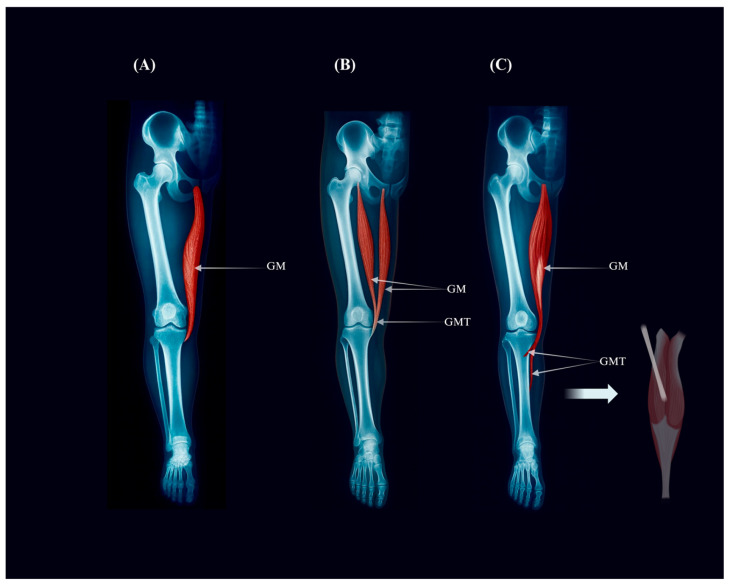
**Morphological variants of the gracilis muscle (IGF Component I).** Radiographic-style illustration showing (**A**) the typical single-belly gracilis (Type Ia), (**B**) the double-headed “gracilis biceps” variant (Type Ib), and (**C**) an accessory-slip variant (Type Ic) extending toward the gastrocnemius. These configurations represent IGF Component I and may influence dissection strategy and flap design.

The principal morphological variants of the gracilis muscle, encompassed within IGF Component I, include the typical single-belly configuration and less frequent double-headed or accessory-slip forms. These structural deviations may modify dissection strategy and flap design. Representative configurations are illustrated in Figure 2.

### 4.2. Tendon Insertion Variants and Pes Anserinus Configuration

Distally, the GM typically joins the sartorius and semitendinosus to form the pes anserinus on the medial tibia. However, tendon insertion is highly variable. Olewnik et al. [11] described six distinct patterns in 102 limbs, including **monotendinous (50–74%), bi- or tri-tendinous insertions**, and frequent **accessory bands** to the crural fascia or gastrocnemius aponeurosis [2,11].

Imaging-based detection is best achieved using high-resolution MRI (thin-slice coronal and axial) or ultrasound, allowing precise visualization of tendon number, insertion points, and accessory bands [2,11,20].

The observed morphological variants of the gracilis muscle and its distal tendon insertions are summarized in Table 1 and Table 2, respectively. These tables provide a practical overview for clinicians, combining anatomical types, frequencies, and their surgical significance.

### 4.3. Clinical and Surgical Implications

A thorough awareness of these morphological variants is of paramount importance for surgical planning. Muscle belly anomalies, such as a double-headed gracilis, can significantly influence the surgical approach, either by providing an unexpected source of additional tissue for reconstruction or by complicating a standard flap harvest if not anticipated [2,11]. Similarly, variations in the pes anserinus insertion are critically important for orthopedic surgeons performing tendon harvesting for procedures like anterior cruciate ligament reconstruction. An unrecognized accessory band can lead to an incomplete tendon harvest or iatrogenic damage to adjacent structures [4,11]. Accurate preoperative identification of these variants through advanced imaging modalities such as MRI or ultrasound is crucial for mitigating intraoperative risks and optimizing surgical outcomes. A structured framework such as the proposed IGF can help harmonise terminology and highlight imaging-traceable variant patterns relevant to planning; however, it has not yet been prospectively validated as a clinical decision rule.

### 4.4. Vascular Variations of the Gracilis Muscle

The GM receives its primary vascular supply from branches of the MCFA and/or DFA, with occasional contributions from the superficial femoral artery [1,3]. A single dominant pedicle typically enters the muscle at its proximal third, accompanied by one or more minor pedicles supplying the mid or distal segments [21].

Cadaveric and imaging-based studies have reported variability in the number and origin of GM vascular pedicles. In most cases, one or two dominant pedicles are observed [1,21]. However, some specimens exhibit three or more sizable pedicles, which can originate from multiple arterial sources, including branches of both the MCFA and DFA [21]. These additional pedicles may enter at variable distances from the pubic origin, altering the perfusion territory and potentially influencing flap design.

Prevalence data from anatomical studies suggest that a single dominant pedicle occurs in approximately 50–60% of cases, two dominant pedicles in 30–40%, and three or more in fewer than 10% [1,21]. Although less common, the presence of multiple pedicles can provide vascular redundancy and permit the design of dual-pedicle flaps, but it also increases dissection complexity and operative time.

Radiological identification of vascular variants is best achieved with computed tomography angiography (CTA) or magnetic resonance angiography (MRA), both of which allow precise mapping of pedicle number, origin, calibre, and intramuscular trajectory [21]. CTA offers superior spatial resolution and rapid acquisition, while MRA provides a radiation-free alternative with excellent soft tissue contrast. Doppler ultrasound (US) can identify superficial perforators but has limited capacity to visualise deep pedicles [1,12].

From a surgical perspective, awareness of vascular variants is essential for safe flap harvest and optimal perfusion. Preoperative identification of multiple pedicles allows surgeons to plan the dissection route, choose the most suitable primary pedicle, and consider dual-pedicle designs in complex reconstructions. Conversely, unexpected pedicle patterns identified intraoperatively may necessitate flap modification or abandonment, underscoring the value of thorough preoperative vascular mapping.

## 5. Innervation Variants of the Gracilis Muscle: The Blueprint for Functional Reconstruction

The innervation of the GM, while primarily supplied by the anterior branch of the obturator nerve, exhibits significant variability that is of profound importance for functional reconstructive surgery. Understanding these neural patterns, both outside and within the muscle, is the key to unlocking advanced surgical techniques such as functional muscle splitting.

### 5.1. Extramuscular Innervation Variants

The extramuscular branching of the obturator nerve to the gracilis muscle demonstrates considerable variability. Kurtys et al. [4], based on a cadaveric study of 85 lower limbs, identified four distinct innervation types, which correspond directly to the IGF classification (Types IVa–IVd).

- Type I (~65%) involves a single dominant proximal nerve entering the muscle near its origin. This pattern is predictable and ideal for standard nerve dissection.
- Type II (~26%) shows a single proximal entry point without accessory branches. It allows for straightforward harvest with minimal variation.
- Type III (~8%) features dual innervation: the main obturator nerve and an additional branch from the nerve to the adductor longus. This configuration permits functional splitting of the gracilis muscle.
- Type IV (~1%) presents a rare dual innervation pattern, where an accessory branch arises from the adductor magnus nerve. This variant provides the highest level of reconstructive flexibility.

These patterns are of critical surgical importance. In particular, dual-innervation types (III and IV) enable the GM to be divided into two independently innervated functional units, facilitating advanced procedures such as dual-target muscle transfers in facial or limb reanimation [4].

**Figure 3:**
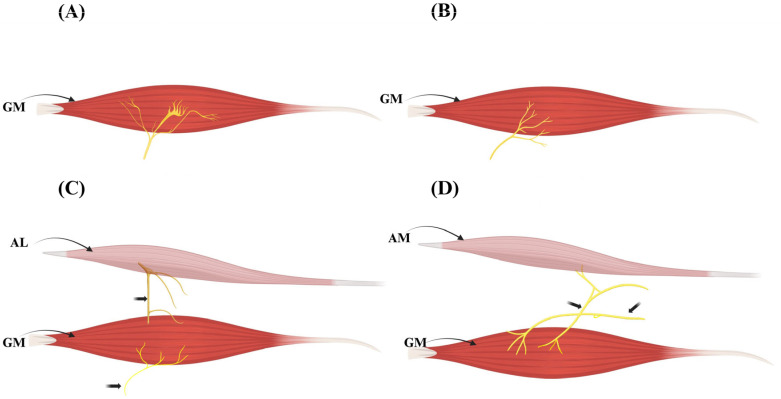
**Extramuscular innervation variants of the gracilis muscle (IGF Component IV).** Schematic representation of the principal extramuscular innervation patterns of the gracilis muscle (GM) according to the Integrated Gracilis Framework (IGF). (**A**) Type IVa—single obturator nerve branch entering proximally near the muscle hilum. (**B**) Type IVb—variant single-branch trajectory with slightly distal entry. (**C**) Type IVc—dual innervation via the obturator nerve and an additional branch from the adductor longus (AL) nerve. (**D**) Type IVd—dual innervation via the obturator nerve and an accessory branch from the adductor magnus (AM) nerve. Dual-input configurations (**C**,**D**) provide the anatomical substrate for functional muscle splitting and selective neurotization in reconstructive surgery.

Representative configurations of extramuscular innervation patterns are illustrated in Figure 3.

An overview of these variants and their surgical implications is presented in Table 3.

### 5.2. Intramuscular Neuromuscular Compartments

The GM exhibits complex internal innervation patterns. Kurtys et al. [9], using Sihler’s staining, identified three distinct types of intramuscular neuromuscular compartmentalization, each with direct relevance for reconstructive surgery and corresponding to IGF Types Va–Vc.

- Type I (~71%): A dominant proximal nerve branch creates clearly defined compartments in the proximal muscle. This pattern supports standard single-function transfers.
- Type II (~24%): Indirect proximal branches originate more distally, resulting in less distinct proximal segmentation. Surgical dissection requires greater precision to isolate functional units.
- Type III (~5–6%): Lacks proximal nerve entry; innervation occurs from mid-muscle or distal zones, often in a retrograde pattern. This variant is highly complex and demands meticulous dissection and imaging.

**Figure 4:**
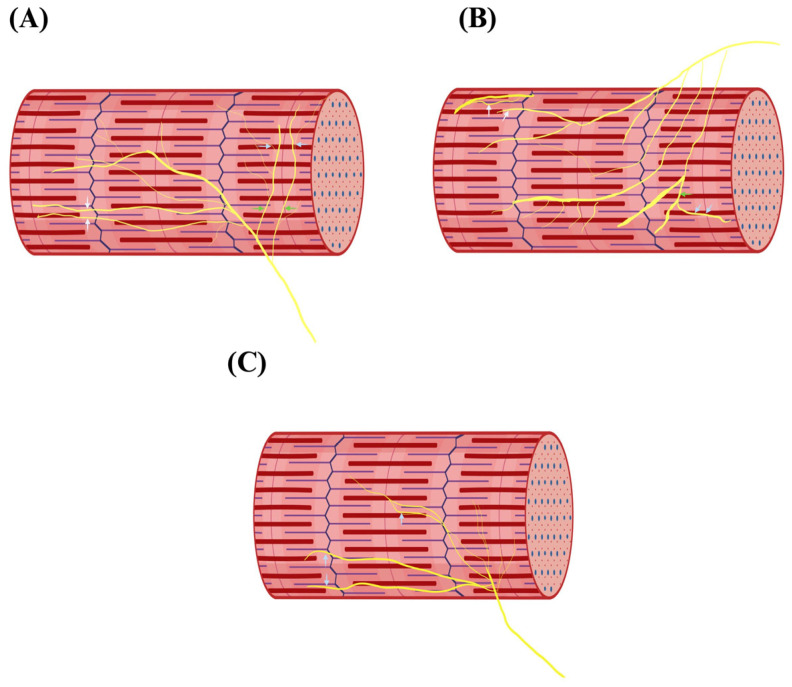
**Intramuscular neuromuscular compartments of the gracilis muscle (IGF Component V).** Schematic representation of intramuscular innervation patterns according to Kurtys et al. [4]. (**A**) Type Va—proximal motor entry forming distinct neuromuscular compartments. (**B**) Type Vb—indirect proximal branching with less defined segmentation. (**C**) Type Vc—distal or retrograde nerve entry supplying the muscle from mid- or distal zones. Yellow lines denote intramuscular motor nerve trajectories. These variants correspond to IGF Component V and determine the feasibility of segmental dissection and selective neurotization.

Representative intramuscular innervation configurations are shown in Figure 4.

These patterns significantly impact functional muscle splitting procedures, where precise knowledge of intramuscular segmentation is crucial. Type III, in particular, may limit the feasibility of segmental transfer without preoperative MR neurography and intraoperative nerve stimulation [9].

An overview of these variants and their surgical relevance is presented in Table 4.

### 5.3. Imaging Visualization of Gracilis Nerve Patterns: The Role of MR Neurography

Although cadaveric studies have revealed the variability of GM innervation, clinical application depends on non-invasive identification in living patients. MRN has emerged as a crucial modality for mapping both extramuscular and intramuscular nerve patterns.

High-resolution MRN, typically performed on a 3T scanner with fat-suppressed T2-weighted sequences (e.g., STIR or SPACE), enables clear visualization of peripheral nerves. It allows radiologists to trace the anterior branch of the obturator nerve through the pelvis and thigh, and to identify motor entry points into the gracilis muscle.

MRN is particularly valuable in detecting:

- Dual innervation patterns (IGF Types IVc and IVd) through visualization of two distinct nerve branches entering the muscle;
- Intramuscular architecture relevant to IGF Types Vb and Vc;
- Indirect markers of neural pathology, such as denervation edema, which appear as T2 hyperintensities.

This modality bridges the gap between anatomical classification and surgical planning, offering a reliable preoperative roadmap for selective neurotization and functional muscle splitting [9].

### 5.4. Surgical Implications: Functional Muscle Splitting and Selective Neurotization

The detailed mapping of gracilis nerve variants especially dual extramuscular innervation (Types III and IV, see Table 3) and complex intramuscular branching patterns (Types II and III, see Table 4) can meaningfully inform reconstructive planning in selected settings. MRN may support non-invasive assessment of extramuscular branching patterns and motor entry points, which can assist preoperative planning alongside intraoperative mapping. The IGF is proposed and not prospectively validated as a clinical decision rule.

In cases of dual nerve entry IGF Types IVc and IVd), the gracilis muscle can be surgically split into two functionally independent segments, each supplied by a separate nerve branch. This anatomical substrate can enable highly specialised procedures, such as:

- Facial reanimation, where one segment is connected to the masseteric nerve (voluntary smile), and the other to a cross-face nerve graft (emotional smile) [7].
- Brachial plexus reconstruction, where one gracilis segment restores elbow flexion while another restores finger extension.

When combined with intramuscular mapping (IGF Types Vb and Vc), surgical teams can ensure flap viability, reduce donor site morbidity, and expand functional outcomes.

These possibilities are summarized in Table 5, which links each anatomical variant with its specific reconstructive utility.

## 6. Advanced Imaging Modalities for Comprehensive GM Mapping

Successful use of the GM in reconstructive surgery benefits from preoperative mapping of muscular, vascular and (where relevant) neural anatomy. Contemporary imaging enables non-invasive characterisation of pedicle number and origin, intramuscular course, perforator distribution and nerve branching patterns, thereby supporting surgical planning and structured reporting. Key modalities, clinical targets and limitations are summarised in Table 6.

### 6.1. Computed Tomography Angiography (CTA)

Computed tomography angiography (CTA) is widely used for preoperative vascular mapping in flap reconstruction when high spatial resolution is required to depict small-calibre pedicles and branching patterns [22,23]. In GM assessment, CTA can delineate dominant and accessory pedicles (origin, calibre, intra- and extramuscular trajectory) and identify anatomical features relevant to flap design and dissection strategy [10,24,25]. Its principal limitations are ionising radiation exposure and constraints related to iodinated contrast administration [25].

### 6.2. Magnetic Resonance Angiography (MRA)

Magnetic resonance angiography (MRA) provides radiation-free vascular mapping and is particularly useful when combined assessment of soft tissues and vascular anatomy is desired [24,26]. Contrast-enhanced MRA can localise perforators, assess vessel calibre and depict intramuscular vascular courses at clinically useful resolution [25]. Limitations include higher cost, longer acquisition times and patient-related contraindications to MRI and/or contrast agents [24].

### 6.3. Doppler Ultrasound (US)

Doppler ultrasound (US) remains valuable as a portable, low-cost modality for initial screening and bedside assessment, enabling localisation of superficial perforators and assessment of gross muscle dimensions [5,20]. Postoperatively, serial measurements may support flap monitoring in selected settings [20]. Limitations include operator dependence, reduced assessment of deep pedicles and lack of comprehensive three-dimensional visualization [5,20].

### 6.4. Magnetic Resonance Neurography (MRN)

Magnetic resonance neurography (MRN) can support planning of functional GM transfers by depicting the obturator nerve course, motor entry points and extramuscular branching patterns, particularly where nerve anatomy influences muscle splitting or targeted reinnervation strategies [2,27]. Its use is limited by availability, cost and the need for high-resolution protocols.

### 6.5. Indocyanine Green (ICG) Fluorescence Angiography

Indocyanine green (ICG) fluorescence angiography provides intraoperative, real-time assessment of perfusion and vascular patency following flap harvest and anastomosis, enabling timely revision when compromised flow is suspected [21,28,29].

### 6.6. Integrative Application of Imaging Modalities

In complex reconstructions, modalities can be used complementarily (e.g., CTA or MRA for vascular mapping, MRN for neural delineation and intraoperative ICG for perfusion confirmation), providing a practical basis for structured reporting and preoperative planning. Where referenced in this context, the IGF should be understood as a proposed, integrative framework rather than a prospectively validated clinical decision rule.

## 7. Clinical and Radiological Implications of GM Imaging

Comprehensive preoperative imaging can refine operative planning for reconstructive procedures using the GM by anticipating variant anatomy and enabling structured reporting. In this clinical context, the Integrated Gracilis Framework (IGF) should be understood as a proposed, integrative framework to harmonise description and planning; it is not yet prospectively validated as a clinical decision rule.

### 7.1. Facial Reanimation

Functional GM transfer for chronic facial palsy benefits from preoperative identification of vascular and neural anatomy relevant to flap harvest, inset and reinnervation. Vascular mapping may reduce the risk of intraoperative pedicle compromise by clarifying pedicle calibre, location and course [22,23,28]. Where nerve anatomy influences muscle splitting or targeted coaptation, assessment of extramuscular branching patterns can support segmentation planning and reduce the likelihood of suboptimal motor targeting [27].

### 7.2. Autologous Breast Reconstruction

In transverse upper gracilis (TUG) flap breast reconstruction, preoperative assessment of perforator distribution and pedicle anatomy supports flap design and may reduce the risk of partial ischaemia or flap loss [22,23,25]. Imaging-derived soft-tissue assessment can additionally inform donor-site selection and expected volume where individualised planning is required [23].

### 7.3. Lower Limb Reconstruction

In lower limb trauma reconstruction, imaging can assist in assessing recipient-vessel suitability and clarifying donor pedicle anatomy within a potentially altered vascular field, thereby supporting operative strategy and risk mitigation [23,28]. Imaging findings consistent with muscle atrophy may also inform expectations regarding long-term functional limitations and aesthetic outcomes [26]. A representative pathway and “red flag” considerations are summarised in Clinical Box 1 and Table 7.

Box 1 **Radiological “red flags” aligned with IGF components (illustrative; IGF is not prospectively validated).**

I. Muscle morphology

Variant example: Double-headed gracilis muscle (illustrative)

Radiological finding: MRI demonstrates two distinct muscle bellies.

Potential clinical risk/pitfall: Misinterpretation as a soft-tissue mass or duplication; inaccurate surgical expectations.

* *

II. Tendon insertion

Variant example: Multiband distal insertion (illustrative)

Radiological finding: Ultrasound reveals multiple distal tendon bands.

Potential clinical risk/pitfall: Incomplete tendon harvest; suboptimal graft length or strength.

* *

III. Vascular pedicles

Variant example: Multiple vascular pedicles (>2) (illustrative)

Radiological finding: CTA or MRA shows a complex pedicle configuration.

Potential clinical risk/pitfall: Increased dissection complexity; risk of perfusion compromise if the dominant pedicle is misidentified.

* *

IV. Extramuscular innervation

Variant example: Dual innervation or nerve communications (illustrative)

Radiological finding: MR neurography demonstrates two motor entry patterns.

Potential clinical risk/pitfall: Incorrect nerve targeting; reduced functional outcome.

* *

V. Intramuscular innervation

Variant example: Distal or atypical intramuscular branching pattern (illustrative)

Radiological finding: MR neurography suggests an atypical intramuscular nerve course (when resolvable).

Potential clinical risk/pitfall: Unexpected postoperative weakness or failed reinnervation if planning assumes a typical distribution.

This box is intended to illustrate potential imaging pitfalls that may align with IGF components. IGF is proposed as an integrative framework and requires prospective validation before clinical decision-making use.

## 8. The Integrated Gracilis Framework (IGF): An Integrative, Clinically Oriented Synthesis

Framing note. The IGF is presented as an integrative synthesis of previously published anatomical, imaging, and surgical classification systems. It functions as a practical crosswalk to align terminology and decision points across domains; it is not a novel classification based on primary data and remains a proposal pending external validation.

This chapter summarises the principles and clinical relevance of IGF, which integrates muscle morphology, tendon configuration, vascular supply, and innervation into a single imaging- and surgery-oriented framework. IGF builds on previously isolated systems and aims to facilitate multidisciplinary communication and preoperative planning. Key components and their clinical implications are outlined in Table 8.

### 8.1. Rationale for a Unified Framework and Limitations of Existing Systems

Despite progress in reconstructive surgery and advanced imaging, current descriptions of the GM remain compartmentalised. Frameworks focusing separately on tendon morphology [11], vascular supply [3,30], and neural architecture [4,8,9,31] have limited integrative value because each addresses only one domain in isolation. This fragmentation hinders comprehensive anatomical correlation, reduces interprofessional communication efficiency, and can limit structured preoperative planning and consistent reporting. An integrated, clinically driven synthesis is therefore useful to consolidate these domains into a single, radiologically traceable and clinically oriented framework intended to support structured planning.

### 8.2. IGF Components (At-a-Glance)

IGF organises five essential domains of the GM muscle morphology, tendon insertion, vascular pedicle configuration, and both extramuscular and intramuscular neural patterns linking each to imaging correlates and surgical implications. The emphasis is on harmonising prior systems and standardising language rather than introducing new categories.

### 8.3. Clinical Application of IGF: Case-Based Example

Scenario: A patient requires dynamic facial reanimation. This case is illustrative and intended to demonstrate the proposed IGF crosswalk; IGF labels are provided for orientation only and the framework has not yet been prospectively validated as a clinical decision rule. Preoperative multimodal imaging and examination yield an IGF profile of Ib–IIa–IIIb–IVc–Va:

- Ib (Morphology): MRI demonstrates a double-headed gracilis (“gracilis biceps”), prompting a modified dissection plan and allowing tailored flap volume [21].
- IIa (Tendon): Ultrasound confirms a monotendinous insertion, facilitating straightforward tendon harvest [11].
- IIIb (Vascular): CTA identifies a dominant proximal pedicle plus a sizable distal accessory pedicle; intraoperatively the team can select the optimal inflow and consider supercharging [32,33].
- IVc (Extramuscular innervation): MR neurography suggests a communicating branch with adductor longus, enabling precise muscle segmentation and potential dual-innervation [4].
- Va (Intramuscular innervation): Proximal-dominant motor point distribution simplifies targeting for functional transfer [9,34].

**Figure 5:**
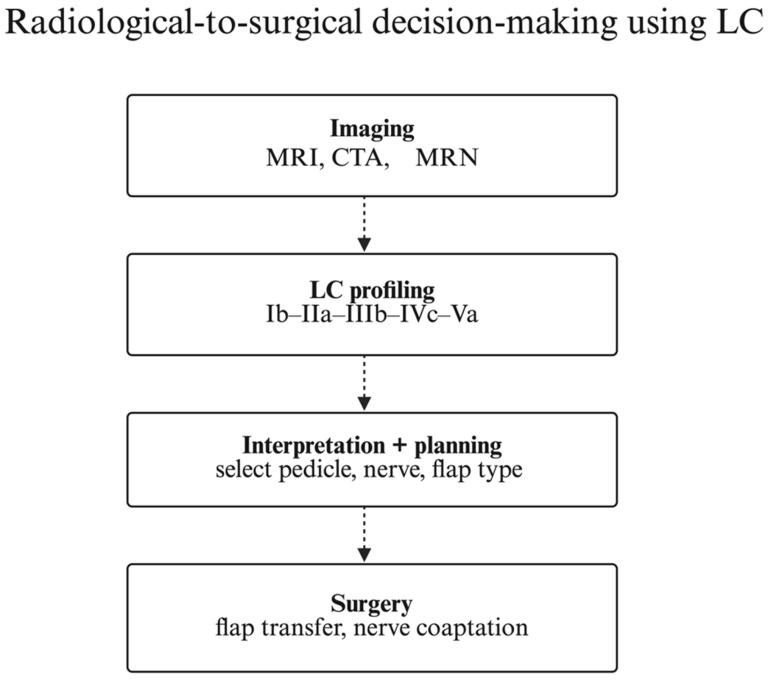
**Radiology-to-Surgery Workflow Based on the Integrated Gracilis Framework (IGF).** A stepwise decision pathway showing how each IGF component (morphology, tendon insertion, vascular pattern, extramuscular and intramuscular innervation) informs imaging choices and surgical strategy. IGF labels are provided as orientation only. IGF is proposed and not prospectively validated as a clinical decision rule.

This anatomy-driven approach aligns radiological findings with operative strategy and may support structured planning and communication; any outcome benefit requires prospective validation. Figure 5 illustrates a radiology-to-surgery workflow that translates IGF components into stepwise clinical actions.

### 8.4. Comparative Context and Need for Validation

IGF synthesises previously fragmented classification efforts into a unified, clinically actionable crosswalk. By integrating morphological, tendinous, vascular, and neural parameters, IGF emphasises radiological integration and a common anatomical language for reconstructive planning.

A structured comparison of representative classification systems, highlighting differences in anatomical scope, clinical utility, radiological integration, and communication clarity, is provided in Table 9.

Unlike earlier systems that focus on isolated structures tendon insertions [2,11], vascular variants [3,30,33], or neural patterns [4,8,9,31], IGF enables cross-domain correlation. However, comparative superiority cannot be inferred from a narrative synthesis alone; prospective validation and standardised reporting are needed (Vila et al. [35] for outcomes harmonisation in facial reanimation).

## 9. Limitations

Despite its integrative design and strong radiological-anatomical foundation, the IGF has several limitations. First, it is currently grounded in existing anatomical studies and cross-sectional imaging data; thus, it requires prospective clinical validation to confirm its predictive accuracy and refine its components based on real-world outcomes [3,24].

Second, significant anatomical variability across demographic and ethnic groups may impact the generalizability of the IGF, emphasizing the need for further population-specific studies [21,25].

Third, advanced imaging modalities central to IGF implementation (e.g., CTA, MRA, MRN) may not be universally accessible. Financial constraints, limited availability, specialist training, and contraindications to contrast agents or radiation exposure may hinder routine clinical use [24].

Lastly, the functional significance of certain complex variants particularly intramuscular neural patterns remains insufficiently defined. Further physiological and electrophysiological investigations are needed to validate their clinical impact [4,8,9].

## 10. Future Directions: Integrating IGF with Imaging and Emerging Technologies

The following directions outline research opportunities to operationalise the proposed IGF; IGF remains nonvalidated and requires prospective clinical evaluation before decision-making use. The IGF provides a robust anatomical framework for improving reconstructive surgical precision. Its next advancement lies in the integration with technological innovations and clinical outcome-based workflows.

### 10.1. Clinical Validation and Functional Correlation

Future studies should correlate IGF profiles with surgical outcomes (e.g., flap viability, complications, functional recovery). Electromyography (EMG) and intraoperative neuromonitoring may further clarify the functional significance of neural variants [3,4].

### 10.2. AI-Driven GM Mapping

Deep learning models such as CNNs and UNETR can automate CTA, MRA, and MRN segmentation, enabling faster, more precise IGF profiling while reducing observer variability [36,37].

### 10.3. Augmented Reality for Surgical Navigation

AR technology, projecting 3D anatomical maps into the operative field, can help identify IGF-based structures intraoperatively, increasing accuracy and reducing surgical time [38].

### 10.4. Tissue Engineering and Regenerative Medicine

IGF-guided reconstructions may support development of pre-vascularized, pre-innervated gracilis grafts customizable and donor-site sparing [39].

**Figure 6:**
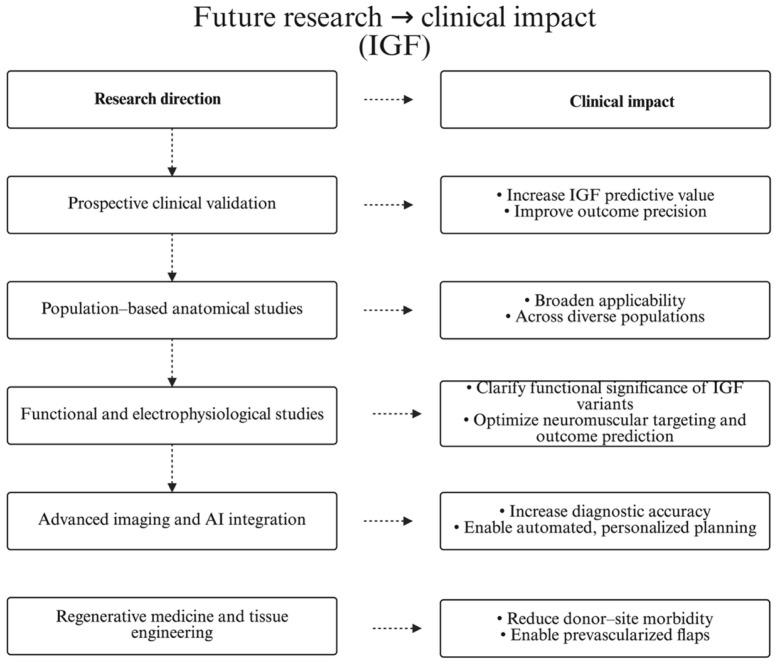
Research → clinical impact (IGF). Each research direction maps to its expected clinical impact; arrows indicate intended effect pathway.

Together, these emerging strategies suggest a paradigm shift in how the IGF framework can be operationalized in clinical and technological settings. An overview of the most promising research avenues and their anticipated clinical relevance is summarized in Figure 6

## 11. Conclusions

This review makes three contributions. First, it consolidates anatomical, embryological, imaging and clinical evidence on the gracilis muscle into a single, structured synthesis. Second, it introduces the Integrated Gracilis Framework (IGF) as a proposed integrative crosswalk that harmonises terminology and aligns morphological, vascular and neural variants with imaging correlates and common pitfalls. Third, it provides a practical preoperative workflow and application-focused summaries to support structured reporting and planning in key reconstructive indications (facial reanimation, elbow-flexion reconstruction, and perineal/breast reconstruction). As the IGF is a nonvalidated synthesis, the next step is prospective validation through cadaver–imaging correlation studies and outcome-based surgical cohorts.

## Figures and Tables

**Table 1:** Variants of Gracilis Muscle Belly Morphology.

Type	Variant	Prevalence	Clinical Significance
Ia	Single muscle belly (standard)	~98%	Predictable dissection
Ib	Double-headed (“gracilis biceps”)	~2%	May confuse identification; offers extra tissue
Ic	Accessory slips (fascia lata/gastrocnemius)	2–7%	Additional flap tissue; increased dissection risk

References: Magden et al. [3]; Rizvi et al. [2].

**Table 2:** Classification of Pes Anserinus Tendon Configuration (adapted from Olewnik et al., [11]).

Type	Tendon Pattern	Surgical Implication
1–1–1 (52.9%)	Classical configuration: one tendon each from ST, GT, and STT	Predictable anatomy; ideal for standard tendon harvesting
1–1–2 (31.4%)	One accessory band from STT	Requires attention during harvest; may extend dissection time
1–1–3 (8.8%)	Two accessory bands from STT	Increased complexity; careful identification needed
1–2–3 (1%)	Accessory bands from GT and STT	Additional structures may complicate tendon isolation
2–1–2 (2%)	Accessory bands from ST and STT; single tendon from GT	Atypical pattern; potential for misidentification during graft preparation
2–2–3 (3.9%)	Accessory bands from all three muscles	Highly variable anatomy; detailed imaging and dissection planning recommended

ST—sartorius tendon, GT—gracilis tendon, STT—semitendinosus tendon.

**Table 3:** Extramuscular Innervation Variants of the Gracilis Muscle.

Type (Kurtys)	IGF Equivalent	Prevalence	Description	Surgical Relevance
I	IVa	~65%	Single proximal entry (±small secondary branch)	Standard nerve dissection
II	IVb	~26%	Single entry, no secondary branches	Predictable harvest
III	IVc	~8%	Obturator + adductor longus branches	Enables muscle splitting
IV	IVd	~1%	Obturator + adductor magnus branches	Allows dual-functional transfer

**Table 4:** Intramuscular Neuromuscular Compartments of the Gracilis Muscle.

Type (Kurtys)	IGF Equivalent	Prevalence	Description	Surgical Relevance
I	Va	~71%	Proximal entry with clearly defined neuromuscular compartments	Standard for single-function transfers
II	Vb	~24%	Indirect branches from more distal origin; less distinct compartments	Requires precise dissection to isolate units
III	Vc	~5–6%	No proximal entry; mid/distal (retrograde) nerve entry	High complexity; detailed mapping needed

**Table 5:** Surgical Utility of Gracilis Neural Variants.

Variant Type	Anatomical Pattern	Assessment Approach	Surgical Opportunity	Clinical Application
IVa	Single extramuscular nerve	MRN—one entry	Standard functional transfer	Basic muscle flap
IVc	Dual entry (Obturator + Adductor Longus)	MRN—two nerves visible	Functional muscle split	Facial reanimation, double nerve coaptation
IVd	Dual entry (Obturator + Adductor Magnus)	MRN—dual nerves (rare)	Advanced flap segmentation	Brachial plexus repair, dual motion
Vb	Indirect proximal intramuscular branches	MRN + intra-op mapping	Segmental dissection possible	Selective innervation for partial transfer
Vc	Distal nerve entry, retrograde supply	MRN (where feasible) + anatomical correlation (Sihler-based evidence).	Complex flap design	Microsurgery with pre-harvest validation

**Table 6:** Advanced imaging modalities for gracilis muscle mapping.

Modality	Target Structure(s)	Optimal Setup	Key Clinical Applications	Strengths	Limitations
CTA	Arterial pedicles, perforators, branching patterns	Arterial-phase thin-slice CTA with 3D reconstruction	Detailed vascular mapping; flap design planning	High spatial resolution; 3D visualisation	Ionising radiation; iodinated contrast constraints
MRA	Arterial/venous pedicles; intramuscular vascular course	Contrast-enhanced MRA or TOF MRA	Radiation-free vascular imaging; combined soft-tissue/vascular assessment	No ionising radiation; good soft-tissue context	Longer scan time; cost; contraindications in some patients
Doppler US	Superficial vessels; muscle dimensions	High-frequency linear probe (e.g., 15–18 MHz)	Screening; perforator localisation; selected flap monitoring	Portable; real-time; low cost	Operator-dependent; limited depth; no comprehensive 3D
MRN	Obturator nerve and branches	High-resolution, fat-suppressed T2-weighted (preferably 3T; 3D where available)	Preoperative nerve mapping; branching/communications assessment	Direct neural visualisation; non-invasive	Limited availability; cost; protocol-dependent
ICG fluorescence angiography	Perfusion of muscle and any skin paddle	Intraoperative near-infrared imaging after IV ICG	Flap viability assessment; intraoperative revision guidance	Real-time perfusion assessment	Intraoperative only; specialised equipment required

Abbreviations: CTA, computed tomography angiography; MRA, magnetic resonance angiography; US, ultrasound; MRN, magnetic resonance neurography; ICG, indocyanine green; TOF, time-of-flight; IV, intravenous.

**Table 7:** Clinical and radiological implications of GM imaging.

Clinical Application	Imaging Modality	Key Radiological Targets	Practical Clinical Implications
Facial reanimation	CTA, MRA	Pedicle calibre, location and course	Supports pedicle planning; may reduce risk of vascular compromise [22,23].
Facial reanimation	MRN	Obturator nerve course; motor entry points; extramuscular branching/communications	Supports segmentation and targeted coaptation planning where nerve anatomy is relevant [27].
Autologous breast reconstruction (TUG flap)	CTA, MRA	Perforator distribution; pedicle anatomy; tissue volume (where applicable)	Supports flap design and donor-site planning; may reduce ischaemia/partial flap loss risk [22,23,25].
Lower limb reconstruction	CTA, MRA	Recipient-vessel suitability; donor pedicle anatomy; indicators of atrophy	Supports risk mitigation and operative strategy; informs expectations regarding function and aesthetics [23,26,28].

Abbreviations: GM, gracilis muscle; CTA, computed tomography angiography; MRA, magnetic resonance angiography; MRN, magnetic resonance neurography; TUG, transverse upper gracilis.

**Table 8:** Integrated Gracilis Framework (IGF)—Components, Typical Patterns, and Practical Implications. IGF is proposed and requires prospective validation.

Component	Type	Description	Approx. Prevalence/Range	Primary Imaging Correlate	Key Surgical Implication
I. Muscle Morphology	Ia	Single muscle belly (typical)	~95–99% across cadaveric series	MRI, US	Standard harvest/dissection.
I. Muscle Morphology	Ib	Double-headed (“gracilis biceps”)/accessory slips	Rare (≤1–3%)	MRI, US	Modified dissection; may alter inset/volume.
II. Tendon Insertion	IIa	Monotendinous (single unified tendon)	~50–75% (varies by series)	MRI, US	Straightforward tendon harvest.
II. Tendon Insertion	IIb	Bi-/tri-tendinous insertions	~20–40%	MRI, US	Careful identification to isolate intended tendon(s).
II. Tendon Insertion	IIc	Accessory fascial bands (e.g., to crural/gastrocnemius fascia)	Variable/uncommon	MRI, US	Risk of incomplete harvest if unrecognised.
III. Vascular Pedicles	IIIa	One dominant proximal pedicle (Mathes–Nahai Type II)	Consistent feature	CTA, MRA	Predictable pedicle dissection; main workhorse.
III. Vascular Pedicles	IIIb	Dominant pedicle with 1–3 accessory distal pedicles	Common variant	CTA, MRA	Option for segmental/”double-paddle” designs; plan mapping.
III. Vascular Pedicles	IIIc	Planned multi-pedicle use (segmental free flap/supercharging)	Selected indications	CTA, MRA	Higher complexity; consider dual outflow.
IV. Extramuscular Innervation	IVa	Single anterior obturator branch entering near hilum	Majority pattern	MR neurography (optional)	Standard motor nerve dissection.
IV. Extramuscular Innervation	IVb	Variant single branch trajectory/entry	Reported	MR neurography	Anticipate altered course during harvest.
IV. Extramuscular Innervation	IVc	Communicating branches with adductor longus (dual-input potential)	Uncommon	MR neurography	Enables functional splitting/dual-innervation concepts.
IV. Extramuscular Innervation	IVd	Communications with adductor magnus	Rare	MR neurography	As above; confirm intraoperatively.
V. Intramuscular Innervation	Va	Proximal-dominant motor point distribution	Most common (Sihler mapping)	MR neurography, Sihler’s	Predictable neuromuscular segmentation.
V. Intramuscular Innervation	Vb	Indirect proximal branching/mixed zones	Reported (minority)	MR neurography, Sihler’s	Define territories carefully before splitting.
V. Intramuscular Innervation	Vc	Distal/retrograde supply emphasis	Rare (~5%)	MR neurography, Sihler’s	Meticulous mapping for reliable innervation.
Legend (component color codes):					
I. Muscle Morphology		II. Tendon Insertion	III. Vascular Pedicles	IV. Extramuscular Innervation	V. Intramuscular Innervation

**Table 9:** Comparative Overview of Classification Systems.

Classification/Study	Anatomical Focus	Clinical Utility	Radiological Integration	Communication Clarity	Limitations	Reference
Olewnik et al. (2019) [11]	Pes anserinus tendon insertion patterns	High (tendon grafting/harvest)	Moderate (MRI/US)	Good	Tendon-only scope	Olewnik et al., 2019 [11]
Magden et al. (2010) [3]	Vascular pedicle patterns	High (flap harvesting)	Good (CTA/MRA/US)	Moderate	Vascular-only scope	Magden et al., 2010 [3]
Kurtys et al. (2023a) [8]	Extramuscular nerve branches	High (nerve management)	Good (MRN)	Clear	Extramuscular nerves only	Kurtys et al., 2023a [8]
Kurtys et al. (2023b) [9]	Intramuscular compartments (Sihler)	High (functional segmentation)	Good (MRN/Sihler)	Clear	Intramuscular nerves only	Kurtys et al., 2023b [9]
Rizvi et al. (2018) [2]	Accessory tendon insertions	Moderate (tendon harvesting)	Moderate (MRI/US)	Moderate	Tendon/fascial only	Rizvi et al., 2018 [2]
Kumar et al. (2024) [21]	Muscle belly morphology variants	Moderate (dissection nuance)	Moderate (MRI/US)	Clear	Muscle-only scope	Kumar et al., 2024 [21]
Integrated Gracilis Framework (present synthesis)	Integrated muscle, tendon, vascular, neural	High (comprehensive planning)	Designed for CTA/MRA/MRN/US linkage	Unified notation	Requires external validation; synthesis of prior work	Present synthesis

## Data Availability

No new data were created or analyzed in this study.
